# Comparative EPR Study on the Scavenging Effect of Methotrexate with the Isomers of Its Photoswitchable Derivative

**DOI:** 10.3390/ph14070665

**Published:** 2021-07-11

**Authors:** Zsolt Preisz, Nóra Hartvig, Balázs Bognár, Tamás Kálai, Sándor Kunsági-Máté

**Affiliations:** 1 Institute of Organic and Medicinal Chemistry, Faculty of Pharmacy, University of Pécs, Szigeti 12, H-7624 Pécs, Hungary; preisz.zsolt@pte.hu (Z.P.); balazs.bognar@aok.pte.hu (B.B.); tamas.kalai@aok.pte.hu (T.K.); 2 Department of General and Physical Chemistry, Faculty of Sciences, University of Pécs, Ifjúság 6, H-7624 Pécs, Hungary; 3 János Szentágothai Research Center, University of Pécs, Ifjúság 20, H-7624 Pécs, Hungary; nora.hartvig@gmail.com

**Keywords:** methotrexate, phototrexate, photopharmacology, photoswitchable, electron paramagnetic resonance (EPR), scavenger

## Abstract

The scavenging effect of the antimetabolite dihydrofolate reductase inhibitor methotrexate (MTX) and the isomers of its photoswitchable derivate, *cis*- and *trans*-phototrexate (PHX), have been compared by ESR spectroscopy, with the application of a cyclic hydroxylamine spin probe. The results showed the most pronounced scavenging effect in the presence of *trans*-phototrexate (*trans*-PHX). At a low concentration (100 µM) *cis*-PHX also showed a greater scavenging effect than the parent molecule MTX. Direct antioxidant properties of the investigated molecules were measured by ABTS scavenging assay, which showed no significant difference between *trans*-PHX and *cis*-PHX, but both of the isomers of PHX showed a higher antioxidant capacity than MTX. These findings imply that *trans*-PHX may have more pronounced anti-inflammatory and tissue-protective effects than MTX, despite the lack of its cytotoxic, antineoplastic effect.

## 1. Introduction

Chemotherapy (the use of cytotoxic agents) is one of the main methods that are applied in cancer treatment, together with radiation therapy, hormone therapy, and surgery. However, the efficacy of chemotherapy is often limited because of the low therapeutic indices and poor adverse effect profiles of these agents [1]. The lack of disease-specific targeting stands in the background of this phenomenon. The most common adverse effects of chemotherapeutic agents are cardiomyopathy, hepatic fibrosis, pulmonary fibrosis, renal insufficiency, nodulosis, lethargy, and fatigue [2,3].

**Figure 1:**
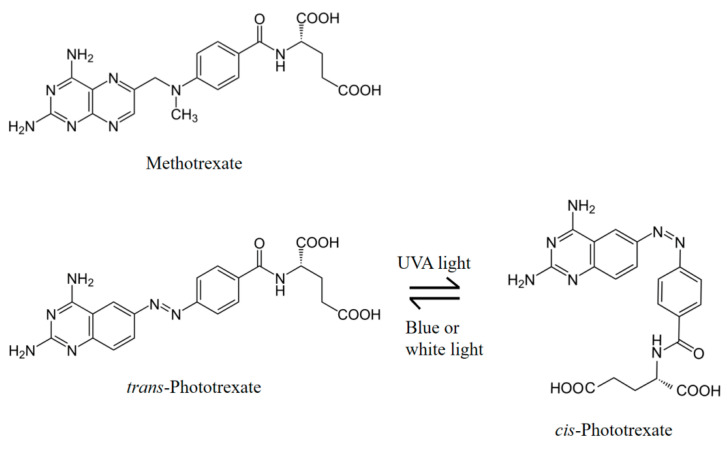
Chemical structures of methotrexate (MTX), *trans*-phototrexate (*trans*-PHX), *cis*-phototrexate (*cis*-PHX), and the reversible isomerization of PHX [6].

Methotrexate (4-amino-10-methylfolic acid, MTX, Figure 1) is an antimetabolite dihydrofolate reductase (DHFR) inhibitor. It is a widely used chemotherapeutic agent in autoimmune diseases such as rheumatoid arthritis (RA), psoriasis, and some sorts of leukaemia [4]. The anti-inflammatory effect of MTX is well-known, but its mechanisms of action are not well understood, several molecular phenomena are described in the literature that can play a role in the development of this effect (inhibition of purine and DNA synthesis, reduction of antigen-dependent T-cell proliferation, promotion of adenosine release, suppression of transmethylation reaction) [2,4,5].

However, MTX has a poor drug safety profile caused by the ubiquity of DHFR [6]. Its most common adverse effects are ulcerative stomatitis, leukopenia, nausea, abdominal distress, malaise, undue fatigue, chills and fever, dizziness, and decreased resistance to infection [7]. Several attempts were made to reduce the side effects of MTX, and some of them have proven to be effective, for example, the concurrent use of leucovorin (folinic acid). This adjuvant does not interfere with the efficacy of methotrexate in a clinically significant manner, but significantly reduces the common side effects of low-dose MTX therapy [8].

To activate the drugs exclusively at their target place of action is a promising approach to improve cancer therapies, and synthetical photoswitches can be suitable tools to achieve this. Photoswitches are chromophores that can be reversibly isomerized when exposed to light. The field that attempts to control biological activity with these molecules is called photopharmacology [9]. Azobenzene is the most widely used photoswitch in biological applications because of the ease of synthesis and functionalization, fast photo-isomerization, and the low rate of photo-bleaching [10,11].

PHX is a photoswitchable azobenzene analogue of MTX that has been synthesized and described by C. Matera et al. [6] and by Mashita et al. [12]. PHX contains a diazene stereogenic unit and its pharmacological activity is higher in its *cis* state than in the more thermodynamically stable *trans* state. It can be effectively isomerized from *trans* to *cis* with UVA light (375 nm) and back-isomerized from *cis* to *trans* with blue (460 nm) or white light (Figure 1). This transition is reversible and can be repeated several times. The antineoplastic effect (and also the adverse effects caused by the cytotoxic activity) appears only in light-exposed regions and decreases in dark regions. Target tissues that can be exposed to UV-illumination are primarily the skin, the digestive, respiratory, and reproductive tracts.

Clinically, MTX reduces the risk of cardiovascular events caused by cardiovascular disease (CVD) which is also recognized as a chronic inflammatory condition [13,14,15]. Modified lipoproteins are assumed to play an important role in the development and progression of CVD. These modified lipoproteins increase oxidative stress and lipid peroxidation, which lead to the increase of reactive oxygen species (ROS) and malondialdehyde–acetaldehyde (MAA) production. The elevated levels of these reactive products have been detected in RA and also in CVD cases [16,17]. It has been described that MTX inhibits malondialdehyde–acetaldehyde–protein adduct formation and scavenges superoxide radicals [18], but these features have never been investigated in the case of *trans*- or *cis*-PHX yet.

**Figure 2:**
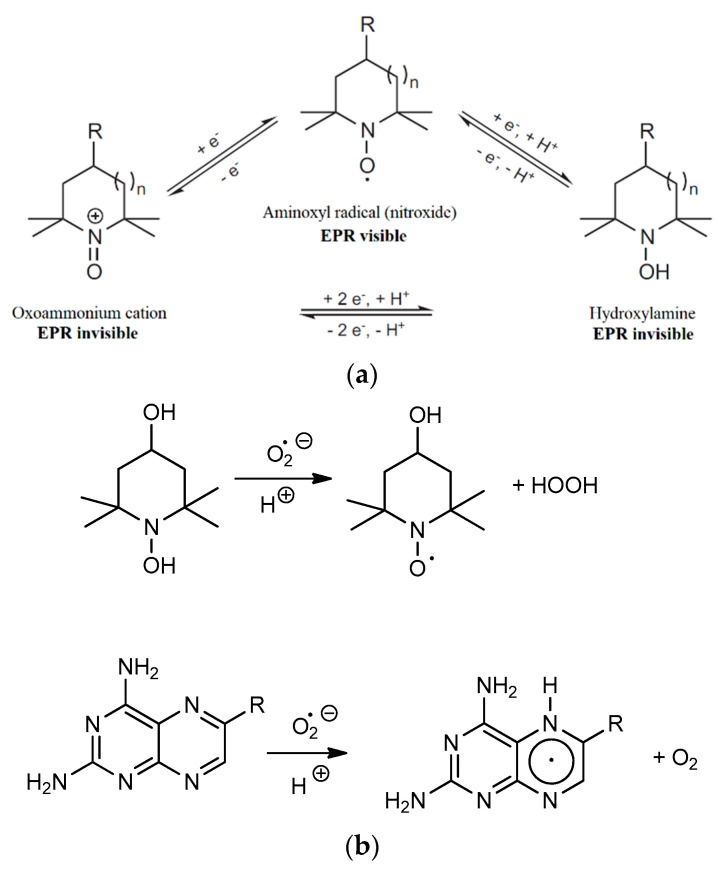
(**a**) (top) Redox species associated with cyclic hydroxylamine spin probe molecules (n = 0 or 1, pyrrolidine or piperidine) [20]. (**b**) (bottom) Proposed redox processes associated with the quenching of superoxide radical by oxidation of hydroxylamine and the reduction of the 1,4-diazine unit of MTX.

EPR spectroscopy has long been a method of choice for the detection of paramagnetic species, such as free radicals or transition metal ions in vitro. EPR spectroscopy in association with molecular probes has been successfully applied to the evaluation of the redox status of tumors or in rodent models of diverse pathological conditions, such as hypertension, stroke, epilepsy, and sepsis [19]. In this study, 1-hydroxy-2,2,6,6-tetramethyl-piperidin-4-ol (*N*-hydroxy-TEMPOL), a cyclic hydroxylamine, was used as a spin probe for EPR measurements. Cyclic aminoxyl radicals with tetraalkyl substituents flanking the N–O^•^ functionality are most often used as probes for the redox status in vivo [20]. Hydroxylamines (in contrast with spin traps) do not have the ability to bind (trap) free radicals. They can undergo oxidation to stable nitroxides, and the nitroxide accumulation can be followed by EPR. Hydroxylamines rapidly react with oxygen-centered free radicals, including superoxide, peroxyl radicals, or peroxynitrite [21]. These stable hydroxylamines can also participate in redox reactions with biological compounds or enzymes and thus report on the redox status of the cell by shuttling between the three forms shown in Figure 2a. Figure 2b summarizes the possible redox process associated with the interaction of the superoxide radical with the MTX and PHX molecules.

By adopting the method established well by Zimmerman et al. [18] we intended to compare the superoxide scavenging activity of methotrexate and *cis*- and *trans*-phototrexate by means of 1-hydroxy-2,2,6,6-tetramethyl-piperidin-4-ol (*N*-hydroxy-TEMPOL), as a $\dot{O_{2}^{-}}$ radical scavenger. The direct antioxidant properties of MTX, *trans*-PHX, and *cis*-PHX, were also investigated by ABTS scavenging assay. PHX is a relatively new molecule, which has already attracted attention [3], but its antioxidant activity has not been investigated before.

## 2. Results

### 2.1. Absorbance Spectra of Trans-PHX before and after EPR Measurements

**Figure 3:**
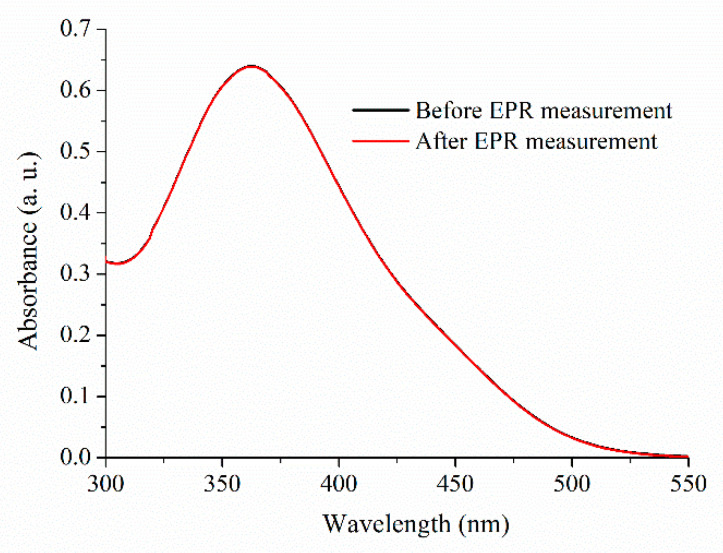
UV-vis absorption spectra of *trans*-PHX samples before and after EPR measurement.

To make sure that *trans*-PHX does not suffer isomerization or other molecular modification as a result of EPR measurements, UV-vis spectra were recorded before and after the EPR measurement (Figure 3). EPR measurements are taken in 30 min. There is no significant difference between the two spectra, so it was found that the 9.1 GHz microwave irradiation of EPR does not cause any change in the *trans*-PHX molecule. The applied concentration of *trans*-PHX was 50 µM.

### 2.2. Isomerization of Trans-PHX

**Figure 4:**
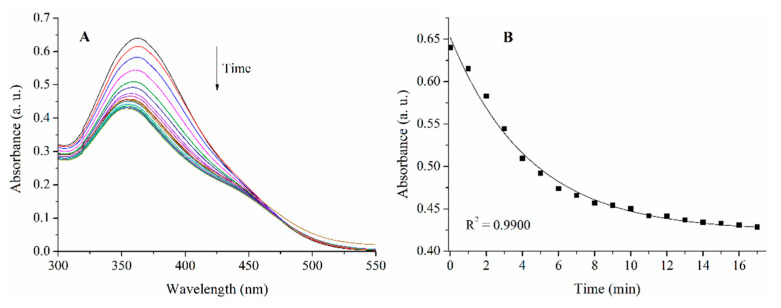
UV-vis spectra of a 50 μM solution of *trans*-PHX exposed to 366 nm light (**A**) and the intensity of the peak at 360 nm plotted against time (**B**). The first spectrum was recorded before the beginning of the UV-light exposure, then measurements were taken every minute.

To investigate the direct antioxidant properties of *cis*-PHX, the complete isomerization of the thermodinamically stable trans-PHX has to be reached. To this purpose UV-light exposure was used (λ = 366 nm). To examine the time dependence of the photoisomerization, a sample containing 50 µM of trans-PHX was exposed to UV-light and the process was followed by spectrophotometrical measurements (Figure 4). According to these measurements, the complete isomerization of PHX (from *trans* to *cis*) was reached after approximately 15 min of UV-light exposure.

### 2.3. Results of EPR Measurements

**Figure 5:**
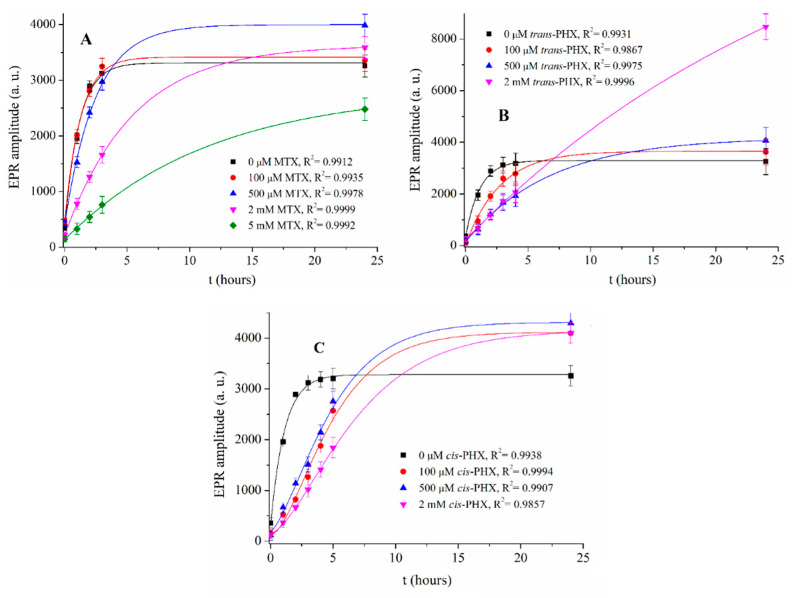
The amplitudes of EPR signals of samples containing 0–5 mM MTX (**A**), 0–2 mM *trans*-PHX (**B**) and, 0–2 mM *cis*-PHX (**C**) plotted against time.

To determine the direct antioxidant properties of MTX, *trans*-PHX, and *cis*-PHX, EPR spectra of samples containing 0 µM, 100 µM, 500 µM, 2 mM, 5 mM MTX and 0 µM, 100 µM, 500 µM and 2 mM *trans*-PHX, and *cis*-PHX were recorded (Figure 5). The average amplitudes of the EPR-peaks were investigated in all cases.

The rate of the production of reactive oxygen species (ROS) has the ability to be very informative, due to the fact that when this rate reaches the saturation level of the biological scavenger systems (e.g., superoxide dismutase, catalase, glutathione peroxidase), the cells will be exposed to oxidative stress. The difference between the maxima of curves is less significant. The reason for the high amplitude values of the sample containing 2 mM *trans*-PHX is not clear yet. The rate of ROS production has not been applied yet for describing the scavenging effect of MTX and PHX. It is observable that the rate of ROS production decreases with the increasing concentrations of MTX and *trans*-PHX, but this effect is more pronounced in the presence of *trans*-PHX. It is important to note that this decrease is not the result of XO inhibition [18]. Based on the curves in Figure 2, the ln k kinetic parameters of all of the samples were determined (Table 1). It was found that *trans*-PHX slows down the ROS production to a greater extent than MTX at all of the investigated concentration levels. This inhibition effect is also more pronounced in the presence of *cis*-PHX when compared to MTX at 100 µM concentration, but at higher concentrations, MTX had a greater effect than *cis*-PHX. This effect is higher in the presence of *trans*-PHX than in *cis*-PHX. We note that the ln k values that were calculated to determine the effect of *cis*-PHX may not be entirely accurate. This is for the reason that the sigmoidal shape of the related curves may reflect for example, the presence of autocatalytic steps. Since the absorption spectra recorded before and after the measurements do not reflect any changes in the *cis*-PHX conformation, further investigations are required to clarify the complex reaction mechanism.

The significance of the scavenging effect of MTX, *cis*-PHX, and *trans*-PHX, has been investigated by performing two-way ANOVA statistics implemented in the OriginLab 8.1 software (OriginLab Corporation, Northampton, MA, USA). The population means related to the concentration of the antimetabolite drugs or their categories (MTX, *cis*-PHX, or *trans*-PHX) were found to be significantly different at the significance level of 0.05 (*p* = 2.59 × 10^−8^ and *p* = 1.710 × 10^−5^ for concentration and drug categories, respectively).

**Figure 6:**
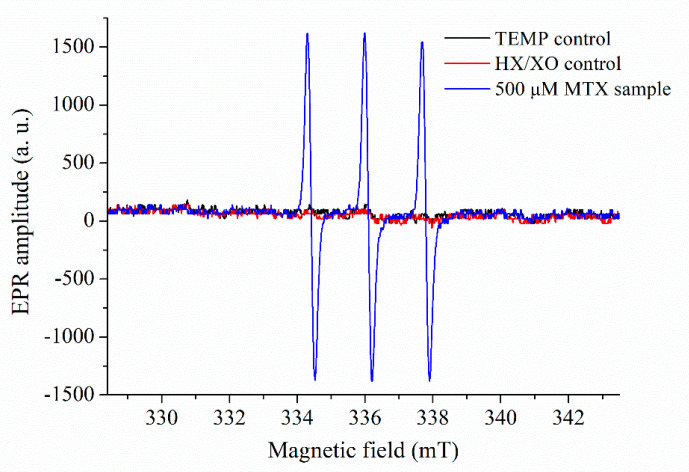
EPR spectra of the controls compared to the sample containing 500 µM MTX. All spectra were recorded 3 h after sample preparation.

Control samples that only contained TEMP solution and HX/XO solution were measured as well, to demonstrate that the spin probe (TEMP) alone does not generate any significant EPR signal, but it is needed to indicate the presence of ROS (Figure 6).

### 2.4. Results of ABTS Scavenging Assay

**Figure 7:**
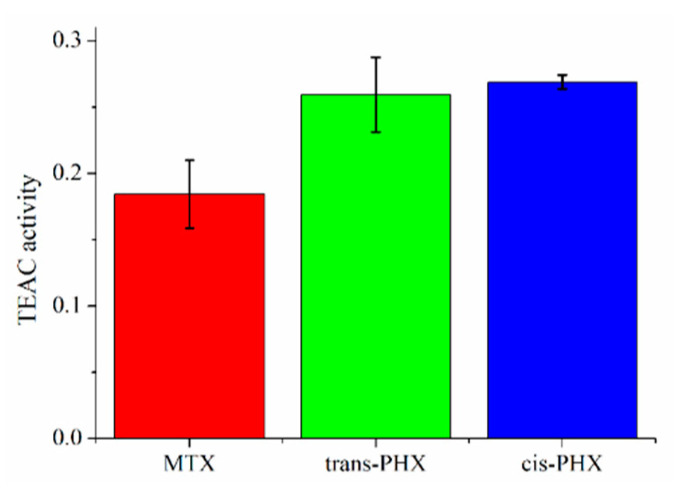
Trolox equivalent antioxidant capacity (TEAC) of methotrexate (MTX), *trans*-phototrexate (*trans*-PHX), and *cis*-phototrexate (*cis*-PHX) measured by ABTS radical scavenging assay. Data represent the mean ± SD (*n* = 3).

The Trolox equivalent antioxidant capacities (TEAC) of MTX, *trans*-PHX, and *cis*-PHX, were determined using ABTS (2,2′-azinobis[3-ethylbenzothiazoline-6-sulfonic acid]) assay, which monitors electron and proton donor activity [22]. The method is based on the green-colored ABTS^•+^ radical, which is detected at 734 nm. Results are depicted in Figure 7.

There was no significant difference between the TEAC values of *trans*-PHX and *cis*-PHX, but both of the isomers of PHX showed a higher antioxidant capacity than MTX.

## 3. Discussion

The role of antirheumatic drugs in cardiovascular disease prevention has been an intensively researched field in the last decade [23,24,25]. Colchicine is the first anti-inflammatory molecule to have been shown in a randomized, double-blind trial to be effective in the secondary prevention of myocardial infarction [26].

Compared with the general population, patients with rheumatoid arthritis (RA) have an increased risk of cardiovascular disease (CVD) or events (CVE) [27] and reduced survival [28]. In patients with RA, treatment with tumor necrosis factor (TNF) inhibitors or MTX was associated with a 30% and 28% reduction in the risk of CVEs, respectively [29]. The ability to scavenge $\dot{O_{2}^{-}}$ appears to be a mechanism by which MTX inhibits the formation of malondialdehyde–acetaldehyde (MAA) adducts. The inhibition of MAA adduct formation and scavenging of free radicals may reduce the inflammation associated with CVD and RA, thereby reducing tissue damage [18].

Phototrexate is a relatively new molecule, therefore very few publications can be found in the literature about it. It has been synthesized and characterized by Matera et al. and its antifolate and antiproliferative properties have been demonstrated by in vitro and in vivo assays [6]. PHX has been derived by azologization and bioisosteric substitutions of MTX. PHX is a photoswitchable molecule, it can be switched on by UV light exposure and switched off by blue or white light or by thermal relaxation in the dark. Its *cis* isomer’s effect is very similar to the effect of MTX, while it is almost inert as an antifolate in its *trans* configuration [6].

In this study, the ROS-scavenging effect of *trans*-PHX, *cis*-PHX, and MTX, was investigated and compared to each other by an EPR spectroscopy method [18]. EPR spectra of *cis*- and *trans*-PHX were recorded and the direct antioxidant properties of these molecules were described for the first time. Kinetic parameters (rate k) were determined and it was found that *trans*-PHX has a more pronounced scavenging effect than MTX and *cis*-PHX. This effect is found to be greater in the presence of *cis*-PHX compared to MTX at lower concentrations. The results of MTX are in line with the study published by Zimmermann et al. [18].

ABTS scavenging assay measurements were also carried out to investigate the antioxidant properties (TEAC values) of MTX and the isomers of PHX. These experiments showed no significant difference between the scavenging effect of *trans*-PHX and that of the *cis*-PHX, but these experiments confirm that both of the isomers of PHX showed a higher antioxidant capacity than MTX.

The reason for the slightly different results of the applied methods is likely due to the methodological difference between these methods. The EPR spectroscopy method is applicable to examine the superoxide radical scavenging activity of the investigated molecules, while by the ABTS scavenging assay the ABTS^•+^ radical scavenging activity was measured.

Despite the slight differences between the two experiments applied here, it can be assumed that *trans*-PHX may have more pronounced anti-inflammatory and tissue-protective effects than MTX. This finding is very interesting because *trans*-PHX lacks the cytotoxic and antineoplastic effects of MTX and *cis*-PHX [6]. Further investigations are required to clarify the mechanism of action of *trans*-PHX and thereby the exact molecular background of this effect.

## 4. Materials and Methods

Methotrexate (MTX), 1-hydroxy-2,2,6,6-tetramethyl-piperidin-4-ol (*N*-hydroxy-TEMPOL), xanthine oxidase (XO, from bovine milk), and hypoxanthine (HX) were obtained from Sigma-Aldrich (St. Louis, MO, USA). *Trans-*phototrexate (*trans*-PHX) was synthesized in our institute according to the scheme published recently [6].

The applied solvent was a Krebs-HEPES EPR buffer consisting of: NaCl (99 mM), KCl (4.69 mM), CaCl_2_ (2.5 mM), MgSO_4_ (1.2 mM), NaHCO_3_ (25 mM), KH_2_PO_4_ (1.03 mM), D-glucose (5.6 mM), HEPES (20 mM), diethyl dithio carbamic diethylammonium salt (DETC, 5 μM), and deferoxamine (25 μM) [30]. The buffer was adjusted to pH 7.4 with HCl. All of the compounds were purchased from Sigma-Aldrich.

A MiniScope MS 200 (Magnettech GmbH, Berlin, Germany) spectroscope was utilized to detect the produced free radicals and to examine the scavenging ability of MTX. The amplitude of the EPR signal is proportional to the number of unpaired electrons present in the sample, facilitating the quantification of free radicals [31]. The amplitudes were determined using the MiniScopeCtrl software.

The following EPR spectrometer settings were applied for all experiments: B0-field: 335.9723 mT, range: 15.0727 mT, sweep time: 30.0 s, modulation: 0.200 mT, and microwave attenuation: 10.0 dB. TEMP was utilized as an EPR spin probe. All measurements were carried out at room temperature (298 K).

To determine if MTX, *trans*-PHX, or *cis*-PHX directly scavenges free radicals, hypoxanthine (HX) and xanthine oxydase (XO) were used (the HX/XO system produces free radicals, primarily $\dot{O_{2}^{-}}$). 1 mL samples were prepared with 100 µM TEMP, 20 µM HX, 10 mU/mL XO and 0–5 mM MTX or 0–2 mM PHX in 1 mL of EPR buffer. 50 μL of the sample was then loaded into a glass capillary tube and inserted into the capillary holder of the EPR spectrometer. Control samples with TEMP solution alone and with HX/XO solution alone were measured as well.

The thermodynamically stable but pharmacologically inactive *trans*-PHX was isomerized with the application of UV-light (λ = 366 nm) provided by a Fluotest lamp (Original Hanau, Hanau, Germany). To ensure the complete isomerization, UV-vis spectra were recorded by a Specord Plus 210 spectrophotometer (Analytik Jena, Jena, Germany). For data collection, the photon counting method with 0.1 s integration time was used and 2 nm bandwidths set and quartz cuvettes with 1.0 cm thickness were applied. The EPR spectra were registered every hour for all of the samples. This measurement lasts 5 min. When *cis*-PHX was measured, the samples were continuously exposed to UV-light (λ = 366 nm) to prevent the isomerization.

ABTS scavenging assays were carried out with the application of a Specord 40 spectrophotometer (Analytik Jena, Jena, Germany). 2,2′-azinobis(3-ethylbenzothiazoline-6-sulfonic acid (ABTS) was solved in phosphate-buffered saline (PBS) that was composed of NaCl (137 mM/L), KCl (2.7 mM/L), NaH_2_PO_4_ (8 mM/L), and K_2_HPO_4_ (1.5 mM/L). The concentration of ABTS was 7 mM. ABTS radical cation (ABTS^•+^) was produced by reacting ABTS stock solution with potassium persulfate at a final concentration of 2.45 mM and allowing the mixture to stand in the dark at room temperature for 16 h before use. For the study of compounds, the ABTS^•+^ solution was diluted with PBS to an absorbance of 0.70 (±0.02) at 734 nm and equilibrated at 37 °C. Stock solutions of MTX, *trans*-PHX, *cis*-PHX, and Trolox (a water-soluble derivative of vitamin E with antioxidant properties) in PBS were added to the diluted ABTS^•+^ solution, in final concentrations of 12.5, 10, 7.5, and 2.5 μM. After this step, the mixtures were incubated for 6 min at 37 °C before measuring their absorbance at 734 nm. All measurements were carried out three times. The percentage inhibition of absorbance at 734 nm is calculated with the usual formula:

(A_0_ − A_antioxidant_)/A_0_ = inhibition (%) (1)

where A_0_ is the absorbance of the diluted ABTS^•+^ solution. The concentration–response curves of the investigated molecules were compared with the curve of Trolox.

## 5. Conclusions

Using cyclic hydroxylamine spin probe the superoxide scavenging effect of the antimetabolite dihydrofolate reductase inhibitor methotrexate (MTX) and the isomers of its photoswitchable derivate, cis- and trans-phototrexate (PHX), have been compared by ESR spectroscopy. The trans-phototrexate (trans-PHX) showed the most pronounced scavenging effect, while at a low concentration (100 µM) cis-PHX also showed a greater scavenging effect than the parent molecule MTX. Trolox equivalent antioxidant capacity (TEAC) values of these molecules have been determined by ABTS scavenging assay. There was no significant difference between the TEAC values of trans-PHX and cis-PHX, but both of the isomers of PHX showed a higher antioxidant capacity than MTX. These findings imply that trans-PHX may have more pronounced anti-inflammatory and tissue-protective effects than MTX, despite the lack of its cytotoxic, antineoplastic effect.

## Figures and Tables

**Table 1:** The calculated ln k values of the samples investigated.

Concentration (µM)	MTX	*trans-*PHX	*cis*-PHX
	ln k	ln k	ln k
0	−8.70 ± 0.16	−8.69 ± 0.18	−8.68 ± 0.15
100	−8.79 ± 0.23	−9.56 ± 0.15	−9.16 ± 0.25
500	−9.46 ± 0.21	−10.46 ± 0.28	−9.19 ± 0.26
2000	−10.25 ± 0.25	−11.96 ± 0.23	−9.51 ± 0.29
5000	−11.01 ± 0.20	-	-

## Data Availability

The data presented in this study are available in the main text.
